# Physically active academic lessons: acceptance, barriers and facilitators for implementation

**DOI:** 10.1186/s12889-018-5205-3

**Published:** 2018-03-06

**Authors:** Sindre M. Dyrstad, Silje E. Kvalø, Marianne Alstveit, Ingrid Skage

**Affiliations:** 1 Department of Education and Sport Science, University of Stavanger, 4036 Stavanger, Norway; 2 Department of Physio-and Occupational Therapy, Municipality of Stavanger, 4068 Stavanger, Norway

**Keywords:** Implementation, Physical activity, Children, School, Fidelity

## Abstract

**Background:**

To improve health and academic learning in schoolchildren, the Active School programme in Stavanger, Norway has introduced physically active academic lessons. This is a teaching method combining physical activity with academic content. The purpose of this paper was to evaluate the response to the physically active lessons and identify facilitators and barriers for implementation of such an intervention.

**Methods:**

Five school leaders (principals or vice-principals), 13 teachers and 30 children from the five intervention schools were interviewed about their experiences with the 10-month intervention, which consisted of weekly minimum 2 × 45 minutes of physically active academic lessons, and the factors affecting its implementation. All interviews were transcribed and analysed using the qualitative data analysis program NVivo 10 (QSR international, London, UK). In addition, weekly teacher’s intervention delivery logs were collected and analysed.

**Results:**

On average, the physically active academic lessons in 18 of the 34 weeks (53%) were reported in the teacher logs. The number of delivered physically active academic lessons covered 73% of the schools’ planned activity. Physically active lessons were well received among school leaders, teachers and children. The main facilitators for implementation of the physically active lessons were active leadership and teacher support, high self-efficacy regarding mastering the intervention, ease of organizing physically active lessons, inclusion of physically active lessons into the lesson curricula, and children’s positive reception of the intervention. The main barriers were unclear expectations, lack of knowledge and time to plan the physiclly active lessons, and the length of the physically active lessons (15–20 min lessons were preferred over the 45 min lessons).

**Conclusion:**

Physically active academic lessons were considered an appropriate pedagogical method for creating positive variation, and were highly appreciated among both teachers and children. Both the principal and the teachers should be actively involved the implementation, which could be strengthened by including physical activity into the school’s strategy. Barriers for implementing physically active lessons in schools could be lowered by increasing implementation clarity and introducing the teachers to high quality and easily organized lessons.

**Trial registration:**

Clinicaltrail.gov ID identifier: NCT03436355. Retrospectively registered: 16th of Feb, 2018.

## Background

Physical activity has been associated with numerous health benefits [1], and most western countries recommend that children do at least 60 min of daily moderate-to-vigorous physical activity. This level of activity has not been achieved for many children and sedentary time for children and youth is increasing [2]. It is thus especially important that school programmes implement physical activity interventions. Increasing children’s physical activity level in school without reducing academic time has been one argument for combining physical activity and academic content [3], so-called physically active academic lessons, or just physically active lessons. Several programmes such as “Energizers” [4], “Take 10” [5], “Physical Activity Across the Curriculum” [6], “Texas I-CAN” [3, 7], “Virtual Field Trips” [8], “Fit and Academically Proficient at School” [9] and “Active Smarter Kids” [10] have introduced physical activity into the school learning environment. Such programmes have shown promising results for children’s physical activity [4, 5, 8], on-task behaviour [4, 7] and academic achievement [5, 6, 9, 10]. Three reviews about physically active lessons have been found, concluding *“Encouraging evidence of improved physical activity and educational outcomes following physically active lessons is provided”* [11], “*Classroom-based physical activity may have a positive impact on academic-related outcomes*” [12], and “*Physically active academic lessons increase physical activity levels and may benefit learning and health outcomes”* [13]*.*

Substantial resources are used to design and test interventions that increase children’s physical activity level in schools. As an example, the research project named “Active Smarter Kids” has provided high quality effect studies and established a webpage where examples of both physically active academic lessons and brain breaks are made available [14]. However, lack of information about factors affecting the implementation of such interventions can prevent useful interventions from becoming part of the school’s daily programme, since translating effective programmes into real life settings is a complicated and long-term process [15]. Process evaluations explore the implementation, reception, and setting of an intervention and aid the interpretation of the outcome results [16]. Process evaluations are important for distinguishing between interventions with no effect, those that are badly delivered and effective interventions that are difficult to implement. Learning from previous successes and failures is therefore important when designing new interventions [17].

The “Active School” programme started in the city of Stavanger, Norway in 2013 and the primary aim was to increase children’s physical activity levels in school. The main intervention component was physically active academic lessons, mainly performed outdoors, with a minimum of two 45-min sessions per week. In addition, one daily 10-min teacher-directed physically active recess, and a daily 10-min physically active homework (e.g., jumping rope, running, strength training), assigned by the teacher, were included as part of the intervention.

After a successful pilot study in 2013–14 [18], a 10-month cluster randomized controlled trial in primary schools was conducted in 2014–15. The effectiveness evaluation showed a tendency for a time × group (intervention vs control) interaction on executive function (F(1, 344) = 3.64, *p* = .057) meaning that increased physical activity in school tended to benefit children’s cognitive functioning even though no effect was found on aerobic fitness [19]. It is not known whether a longer intervention period would have resulted in significant effects.

Recently, Norwegian politicians decided to prepare a work programme for the implementation of 60-min daily physical activity for all children in grades 1–10. Gleaning information about implementation factors such as teachers’ and pupil’s response to these lessons would be tremendously helpful when deciding whether physically active academic lessons would be an appropriate tool to implement in the regular school curriculum.

Implementation factors specifically related to teacher implementation of classroom physical activity have been identified [20–23]. However, little focus has been given to identifying barriers and facilitators when implementing outdoor physically active academic lessons, and efficient implementation strategies are missing from the literature to date.

The present paper reports findings from a process evaluation embedded within the “Active school” randomized controlled trial, evaluating the implementation of physically active academic lessons with a focus on the following research questions:

1. What were school leaders’, teachers´ and children’s responses to the physically active academic lessons?
2. What were the facilitators and barriers to implementing physically active academic lessons?

The work of Fullan [24] was used to identify other important implementation factors and are specified in Table 1. The school leaders were either principals or vice-principals.

**Table 1:** Interactive factors affecting implementation (Fullan [24])

Characteristics of change	Local factors	External factors
Need of change Clarity about goals and means Complexity: the difficulty and extent of change required to those responsible for implementation Quality and practicality of the programme	School district Community Principal Teacher	Government and other agencies

## Methods

### Design and participants

The Active School Study 2014–2015, was conducted in Stavanger, Norway and ran from Aug 2014 to June 2015. Intervention period lasted 34 weeks. The participants were 483 children in the fifth school year (aged 9–10) from nine schools that were randomly assigned to four control schools and five intervention schools. This age group was selected since effective physical activity school programs for this age group are sought after that may limit the documented physical activity reduction in Norwegian children between ages 9 and 15 years [2]. Eleven school classes participated in the intervention group (*n* = 227), and 17 teachers conducted the intervention. The number of children from each school ranged from 19 to 80, and the size of the classes varied from 19 and 26 children. The participating schools were from both urban and suburban areas of the city. The schoolyards consisted mainly of asphalt and gravel surfaces, ball fields and playground equipment such as slide, sandbox and swings. As far as we know, none of the school leaders have previously participated in similar interventions. Since only four of the 17 teachers in the intervention schools were physical education teachers, the majority of teachers were unfamiliar with organizing children in physical activities.

A total of 90% of all children (227 of 251) and their parents gave their written consent to participate in the data collection. All children were included in the intervention. Children who did not give their consent, were not tested in the effect study or interviewed in the present study. Teachers and school leaders gave their verbal consent to participate in the interviews. The study was approved by the Norwegian Social Science Data Services (project number 38509).

### Teacher training

Fifth-grade teachers in the intervention schools delivered the intervention. To assist and support teachers in conducting the intervention, intervention schools received one primary and secondary contact from the “Active school” project team who throughout the school year regularly attended meetings with the participating teachers. The school contacts were four physiotherapists and one teacher who assisted teachers in the implementation procedures, provided examples of physically active lessons and discussed pedagogical experiences and challenges. To train and support the intervention teachers, one pre-intervention seminar and one midway-seminar were arranged. The pre-intervention seminar presented the rationale for the intervention and provided examples of ways to organize the intervention. At the midway seminar, each school was responsible for a 10-min practical teaching session with 5th grade children performing outdoor physically active lessons. This was followed by a discussion about pedagogical suggestions and reflection on how to improve the content in the lessons (e.g., increase intensity; increase the variety of the academics and physical component of the activity). Content for new physically active academic lessons were shared between intervention schools through a webpage [14]. Intervention schools were provided with equipment such as jump ropes, balls and various other materials such as scrabble letters and cubes.

### Intervention

Since the intervention is fully described elsewhere [19], a brief overview of the physically active academic lessons is provided. At least two 45- min physically active academic lessons should be delivered every week. These lessons were mainly carried out in the schoolyard, and could be integrated in any school subject at the curriculum. A common language activity was Scrabble relay where children worked in groups. Two children from each group ran to a box containing laminated letters, picked one letter, ran back and alternated. The letters were put together in words across and down in English or Norwegian. A popular math activity was Bingo, where each group received one Bingo board. Laminated sheets with math tasks were placed in the schoolyard. The groups ran to the sheets, solved the task, and ran back to the teacher with the answer. If correct, teacher placed a cross on the Bingo board, instructed them how they should move to the next post. They could run forward/backward/in pairs, jump, hop on one foot or move like different animals. If they answer incorrectly, they could try again, with or without the textbook. The teachers developed and shared new physically active lessons during the school year. The academic focus was on repetition, and memorization of knowledge learned in an earlier class.

### Data collection

#### Interviews

Since assessment at a single point in time is unlikely to provide a true estimate of implementation [25], interviews were performed at two points in time. To study the start-up of the intervention, the first interviews were conducted eight weeks into the intervention. They included five group interviews involving two to four teachers at each intervention school. All teachers were positive to participate, but some were inaccessible. In total participated 13 of the 17 intervention teachers (both men and women, age 25–55 years) and the interviews lasted on average 37 min (range: 33–42 min). In addition, school leaders at schools 3 and 4 were separately interviewed, with interviews lasting 11 and 15 min, respectively. Following completion of the intervention, two group interviews at every intervention school were conducted: one for teachers (13 of the 17 intervention teachers in total, these were the same as during the first interview with only a few exceptions) and one for children (six children at each intervention school). Teachers were asked to invite children of both genders, and children with high/low skills in both physical education and academic performance. Five individual interviews for the intervention school leaders, three principals and the two vice-principals who served as the schools project manager, were also conducted. Group interviews for teachers and children were designed to create a discussion around the questions and to limit the time used for data-collection. The interview guide during the second interview round was based on the first, but adjustments were made according to answers from the first round. Average interview times were: 39 min for teachers (range: 30–55 min), 27 min for children (range: 24–32 min), and 26 min for each school leader (range: 20–35 min). Topics in the semi-structured interview guide were based on the work of Fullan [24] and included, for example, information and support, expectations, motivation and experiences from the intervention. Examples of questions that were asked at the first interviews (eight weeks into the intervention) were: How was your motivation for the intervention? What do you think about the information prior to start of the project? What should be changed in the continuation of the intervention? Examples of questions following completion of the intervention (after 34 weeks) were: How have you experienced the facilitation for the planning and implementation of physically active academic lessons? What barriers concerning the implementation have you met? What is important for you in order to continue using physically active academic lessons?

#### Teacher reports

Teachers involved in the intervention were asked to complete a weekly digital log describing content, duration and intensity used every week for physical education, physically active academic lessons, teacher-directed physically active recess and physically active homework. Estimated time to complete the weekly log was 2–3 min, and the logs were sent by email to school’s primary contact every fourth week.

### Data preparation and analysis

Data from the teacher reports were entered in Excel, where descriptive statistics (means, standard deviations, and percentages) for each activity at each school were calculated. All interviews were digitally audio recorded, transcribed in full and analysed using NVivo 11 (NVivo qualitative data analysis software; QSR International Pty Ltd., London, UK).

Qualitative data were analysed using a qualitative content analysis [26]. The analysis consisted of an iterative process. Text from interviews was divided into meaning units that were condensed and coded by two of the authors using an inductive approach [27]. Similar events and experiences were thereafter labelled and grouped into categories and subcategories. In the last step of the analysis, all authors discussed themes and shared ideas, which contributed to a more complete understanding of the data, inspired by the theoretical framework by Fullan [24] treating factors affecting implementation in schools (Table 1). The following factors were included: perceived need for the intervention and experiences from the intervention; clarity about goals and means; complexity of the intervention and the schools’ capacity to implement it; quality and the practicality of the implementation (relative to the factors of need, clarity and complexity); support and children’s response.

Due to financial limitations of the present study, the external factors and some of the local factors in Table 1 were not included in the evaluation. The interviews were conducted in Norwegian and selected quotes were translated into English after the analysis. The themes are presented under separate headlines in the results and illustrated by selected, anonymised quotes that typify the data from interviews.

## Results

### Implementation fidelity

The implementation fidelity describes the extent to which the physically active academic lessons were delivered as planned. On average, the physically active academic lessons in 18 of the 34 weeks (53%) were reported. Two teachers did not use any teacher logs during the intervention period. One school missed only one week of logging, while the rest of the schools submitted around 50% of the teacher logs (Table 2). The number of delivered physically active academic lessons is shown in Table 2, and covered 73% of the schools’ planned activity (mean of the five fidelity numbers), which were 90 or 135 min/week of physically active lessons.

**Table 2:** Number (%) of weekly teacher logs received and reported minutes (min) of weekly physically active academic lessons at the intervention schools during the 34-week intervention

School	Log delivery Number of weeks (% of 34 weeks)	Weekly amount of planned physically active lessons	Delivered min of physically active lessons Mean min/week (SD)	Fidelity in %
1	33 (97)^a^	3 × 45 min	90 (44)	67
2	14 (42)^c^	2 × 45 min	45 (0)	50
3	18 (55)^a^	2 × 45 min	81 (26)	90
4	16 (48)^b^	2 × 45 min	71 (7)	79
5	18 (55)	3 × 45 min	109 (9)	81

^a^2 teacher logs

^b^3 teacher logs

^c^1 of 3 teacher logs

Four of the intervention schools scheduled the physically active lessons at least four weeks in advance, while school 2 did week-to-week planning and delivered 50% (fidelity) of the physically active academic lessons (Table 2).

### Interviews

#### Need and experiences

Both at the beginning and at the end of the intervention, all principals and most teachers were positive to the intervention. One teacher expressed it this way:

*We were positive to the project because the children need to be physically active. Physical activity provides a break to the indoor sitting. I think children are able to achieve more because they are physically active.*

Teacher 2 at school 3.

However, there was an example at one school, where teachers’ motivation varied greatly:

*I have faith in the importance of the project.*

Teacher 2 at school 1.

*I have to admit … that I do it because I have to.*

Teacher 3 at school 1.

For the principal at school 1, the main motivation to participate in the project was to change the school culture from an individual culture where teachers worked as isolated individuals, to a more cooperative culture where teachers exchange ideas and support each other.

Another school leader said:

*We want to work research-based at this school and we know how important physical activity is for both mental health and learning.*

School leader at school 4.

Outcome expectations were that the physically active academic lessons would contribute to a more varied and less sedentary school day, improve children’s health and academic performance, and improve concentration and well being among the children.

Experiences from the physically active academic lessons were highly positive. The teachers expressed that physically active academic lessons were an appropriate pedagogical method, particularly for repetition, but also for learning new topics.

*The children found it easier to remember the multiplication tables when they were out running. They build teamwork skills and they do repetition in a different way. So I’m sure there’s an academic effect of the project.*

Teacher 1 at school 1.

Teachers reported that physically active academic lessons created a positive variation during school day.

*…it’s fun for the children. It (physically active academic lessons) is different than the classroom theory they are so used to, and it provides variation. And it motivates me too, since it gives me the opportunity to get outside.*

Teacher 1 at school 5.

Several teachers noticed that children with poor motor development and low aerobic fitness improved both their motor skills and fitness.

*…I see some of the children who struggled most during the fall, they have now become really fit when we see them run uphill.*

Teacher 1 at school 1.

Classes at schools 3 and 4 included some challenging children, and their teachers experienced a better class environment and saw that the children were able to focus for longer periods after introducing physically active academic lessons. Other teachers did not experience any change because their classes’ work environments were not a problem.

Several teachers said that academically disadvantaged children worked better in groups during the physically active lessons because they were supported by the academically strong children. This seemed to be motivating for all children’s learning.

All the interviewed teachers wanted to continue to use physically active academic lessons in some ways, but wanted them to be less frequent, unscheduled and of a shorter duration.

#### Clarity

Project information was given to both school leaders and teachers. While teachers at three of the five intervention schools thought the project information and the provided training were satisfactory, teachers at two intervention schools thought they had been given too little and unclear information. A school leader at school 3 stated:

*I noticed that I perhaps could have been even clearer to the staff when it came to information at the start.*

Several teachers expressed that there had been too many people involved in the information process, and that not all aspects of the project were clarified. Some teachers expected that the external interventionists would carry out the intervention and not themselves, and felt that the intervention training was unclear and insufficient, which lowered their motivation. The same teachers also expressed that clarity improved when one external contact person was appointed.

*At the beginning there was a lot of information, and some of the information was unclear until we got one contact person.*

Teacher 1 at school 1.

Two workshops were arranged (October and January), and teachers expressed that the workshop in January clarified how to perform physically active academic lessons.

The principal at school 5 said that it would have been easier to follow up the teachers if a detailed intervention plan for the whole school year had been provided at startup. The leaders at schools 1 and 4 mentioned that it was unclear what the teachers should register and report in the log, while the principal at school 3 said that information regarding the amount of external teacher support was imprecise. The school leader at school 2 had no comments regarding the project’s clarity.

#### Complexity

Most of the teachers felt that the complexity of the physically active academic lessons was manageable, but noted that it was time consuming and difficult to plan and develop physically active academic lessons of high quality.

*It would be have been a lot easier to carry out physically active academic lessons if we had received pre-planned physically active academic lessons, but at the same time it should fit into the part of the subject they (the children) are doing there and then. I realize that this would not have been easy to arrange, but it would have made it easier.*

Teacher 1 at school 2.

Teachers at schools 1, 3 and 5 felt they lacked competence at the startup. During the intervention period, teachers’ sense of achievement increased, which strengthened their involvement and self-efficacy, enabling them to plan and carry out the intervention.

*What I found difficult was how to combine physical activity and subjects. There was also a large difference preparing a regular classroom lesson and an outdoor physically active academic lesson…. but gradually such teaching plans (for the active academic lessons) has become easier to prepare.*

Teacher 3 at school 1.

*As we began to understand how to plan and organize physical activity and academic lessons, it became just like a normal lesson. The children know what to do, it’s on the curriculum, it’s just like a regular lesson… It’s about developing a sense of ownership to the method, instead of adopting somebody else’s ideas.*

Teacher 1 at school 3.

The same teacher also mentioned one factor related to the intervention and children needing special care:

*Those who have social difficulties need a thorough explanation of what to do.*

#### Quality and practicality

The teachers had many competing demands during the school day and several teachers mentioned lack of time for planning the physically active lessons as the biggest challenge. Completing the teacher logs was also an extra task during the intervention period.

Teachers at school 2 had a challenge when performing the physically active academic lessons in the schoolyard since classes at the elementary school had recess at the same time. Teachers at school 4 had difficulties filling 45 min with physically active academic lessons.

Other teachers expressed that the following factors strengthened the quality of the implementation: extra time granted by the principal to plan the physically active academic lessons, inclusion of the physically active academic lessons into the weekly schedule, and loyalty to the principal’s expectations of implementing the intervention.

One teacher emphasized children’s positive reception:

*The fact that the kids think it’s fun, motivates us to make exciting tasks.*

Teacher 3 at school 1.

The importance of availability and quality of equipment such as waterproof blotting pads and laminator, to perform outdoor education in rain and wind, was also highlighted and one teacher expressed it like this:

*It is easier to be motivated (for preparing physically active lessons) when you know that the practical equipment is in place.*

Teacher 1 at school 1.

The principal at school 1 emphasized the importance of gradual implementation of the intervention:

*Since we are not obligated to provide this intervention, we have to progress gradually, succeed and build competence one step at a time.*

#### Support

All teachers at the intervention schools felt they received support from the schools’ leaders. However, each principal’s involvement varied from passive to active. While one principal delegated the follow-up to the vice principal, another principal was personally involved and participated in information meetings. Some teachers were given extra resources to implement the intervention, while teachers at other schools received no additional privileges. Several teachers also expressed, separately, that follow-up by the school leaders was insufficient.

*What I lacked from the school leaders was involvement, that they had observed what we were doing and asked how things were going.*

Teacher 3 at school 1.

None of the interviewed school leaders had participated in the physically active lessons together with the teachers. One of the principals acknowledged that this had resulted in a lower knowledge, ownership and involvement in the project.

*I should have been closer to the teachers, I was not aware of how much support they felt they needed. I know that they have requested that I join them out in the schoolyard, I’ve seen it through the window, so I’m fully aware all the activity that is out there.*

School leader at school 1.

Several of the teachers emphasized the importance of the cooperative teamwork.

*The reason we succeeded was because of our cooperation. We are good at different things, and we have helped each other.*

Teacher 1 at school 1.

The importance of programme champions was also highlighted as a motivating factor for planning and implementation. One external contact person per intervention school was established to facilitate the intervention. Even though schools 1 and 2 expected more help to carry out the intervention, all teachers felt that the external support was important for the implementation process, and that these contacts were helpful and supportive.

*If we have questions, we just send an email and get an answer (from the external contact) right away.*

Teacher 1 at school 3.

#### Children’s response to the intervention

Two months after startup, teachers performing the intervention were asked how the children had responded to the intervention. Their general impression were that most children liked the active school lessons very much.

*The children ask what lesson they should be physically active in today.*

Teacher 1 at school 1.

During the interviews at the end of the intervention, children expressed their experiences related to the physically active academic lessons as “fun”, “enjoyable”, “fantastic”, “like”. The children were asked what they thought was the most fun about the physically active academic lessons. Some children emphasised “being physically active”, others highlighted “being outside”, and some liked working with the different tasks they were given. For some children it was important to win, others expressed the sense of achievement, while several children mentioned that they liked to work in groups.

While different types of physical games were successfully included in the physically active lessons at school 2, some of the children at school 5 wanted more variation during the physically active academic lessons.

*There’s too much running because that’s really the only thing we do.*

Child 18 at school 4.

Some children said they had become more physically active during the year and felt an increased aerobic fitness. Children at four out of the five intervention schools said they liked the physically active academic lessons because it added variation to the school day.

*I think it is fun with a lot of physical activity, I would like to do it even more because it’s much more fun than just sitting inside and writing.*

Child 10 at school 3.

Some children mentioned that tasks in the physically active academic lessons had not been challenging enough and contained too much repetition.

*It was the same calculations over and over, and I don’t like that very much since we know them by heart. I would like to have more challenging multiplication tasks.*

Child 23 at school 1.

## Discussion

The main findings of the study were that school leaders, teachers and children considered physically active academic lessons to be a meaningful and feasible teaching method in school, adding variation and fun to learning. Active leadership, positive outcome experiences, teacher support, high sense of achievement and inclusion of physically active lessons into the weekly schedule facilitated the implementation. The main barriers to success were lack of clarity, lack of planning time and insufficient intervention training for the teachers.

Teachers reported that 73% of the planned physically active academic lessons were delivered. This was higher than for teacher-directed physically active recess (which was a minor and less complex part of the present intervention, data not shown). Both teachers and children expressed a higher need for physically active lessons than active recess since many children were already physically active during recess. Compared to other studies [17, 28], the intervention fidelity in the present study was high and could be explained by the close follow-up and guidance by the external interventionists.

Time spent in physically active lessons varied from 45 to 109 min/week between the intervention schools. This illustrates the reality when it comes to implementation in schools. Teachers have different levels of motivation for changing their teaching methods and daily routines, and schools have different leaders and premises for implementing the intervention. The two schools with the highest mean minutes of physically active lessons often performed three weekly physically active lessons since they had one fewer weekly physical education session than the other schools. Regardless of the amount of time spent on physically active lessons, both teachers and children had highly positive experiences with this teaching method, and the interviews revealed both facilitators and barriers affecting the implementation of physically active academic lessons.

### Facilitators

Implementing an intervention that both teachers and school leaders characterized as beneficial seemed essential. This created positive outcome expectations that provided motivation to implement the lessons. Positive attitudes and beliefs regarding physical activity are found to serve as a motivational resource for prioritizing classroom physical activity [22]. Thus, a careful examination of whether or not the intervention is perceived to be important and beneficial seems important. Others have s also highlighted this statement [24, 29].

Principals deciding to take an active leadership role in the intervention got more involved in the project than principals who undertook a more passive leadership. Active leading principals facilitated the implementation by adjusting the workload for the intervention teachers and maintained focus on the intervention. However, even principals performing active leadership were not aware of the significance that even small acknowledgements to the intervention teachers could have. Leadership motivation and engagement is found to be an important implementation factor [30, 31]. As stated by Durlak and DuPre [15]: *Leadership is important in terms of setting priorities, establishing consensus, offering incentives, and managing the overall process of implementation.* The support of administrators and other teachers is found to be associated with teacher implementation of structured classroom physical activity [21, 22].

Among teachers, a high self-efficacy or perceived competence regarding mastering the intervention was important for the implementation. Teachers who established good routines for the planning of physically active academic lessons expressed that these lessons became less demanding with experience. Other studies have reported that perceived competence in integrating classroom based physical activity could be an important predictor of teachers’ use of classroom physical activity [32], and that classroom management skills related to classroom physical activity was a barrier [23]. To rapidly improve teachers’ self-efficacy, we recommend instructing the teachers in easily organized physically active lessons, providing support to teachers for developing their own effective physically active lessons and facilitating a cooperative climate among teachers. This seemed essential for those teachers who developed a sense of ownership of this teaching method. It resulted in a positive reception from the pupils, which again increased teachers’ motivation for the intervention. The importance of proper training of the interventionists is also emphasized in other studies [15, 30, 33, 34].

Most teachers identified the inclusion of physically active lessons into the subject curricula as a facilitator. The teachers felt committed to using the physically active lessons in their weekly teaching, which seemed important for increasing teacher confidence with the method. An obligation and permission to devote class time to physical activity was also found to be a facilitator in the study by Naylor, Macdonald, Zebedee, Reed and McKay [35]. Webster, Zarrett, Cook, Egan, Nesbitt and Weaver [22] reported that scheduling physical activity into daily routines reflected the teachers’ ability to truly integrate, rather than add, physical activity into classroom life. Before teachers have the ability to integrate such physical activities in daily school life, it seems wise to include physically active lessons into the subject curricula to strengthen the obligation to the intervention.

### Barriers

During the early months, most teachers found it both difficult and time consuming to plan good and varied physically active lessons. Several teachers identified lack of time as one of main barriers to implementation fidelity. Fullan [24] emphasize that mastering new routines takes time, and it could important to prepare teachers for a difficult start. Since lack of time is found to be the factor most consistently identified as a barrier to the implementation of school-based physical activity initiatives [29], careful consideration of actions that lower teacher overload and competing demands in the start-up phase seems important. Availability of resources to support implementation is found to have an additive association with implementation of structured classroom physical activity [21], and could be an effective way to lower the ‘lack-of-time’ barrier.

Teachers at two of the five intervention schools expressed that information and expectations given prior intervention start were insufficient, unclear, and were given by too many different people. This resulted in a decreased motivation among some teachers. Lack of clarity, diffuse goals and unspecified means of the intervention are recognized as a major problem in the implementation stage [24]. One external contact person was therefore assigned to every intervention school to provide information and support to the teachers. This was well received. However, some teachers expected and needed a lot more practical help with the intervention than others, showing that schools needed individually adapted information and help.

While four of the intervention schools scheduled the physically active lessons at least four weeks in advance, one school did week-to-week planning. This provided more freedom but resulted in the least amount of reported physically active lessons of the intervention schools. Teachers at this school said they often forgot about the physically active lessons, and that they lacked suitable outdoor areas for conducting physically active lessons due to recess for younger children. Not including physically active lessons into the subject curricula was therefore a barrier for implementation.

The physically active lessons lasted 45 min. Several teachers found it difficult to conduct active lessons for such a long time period, and would have preferred a duration of 15–20 min. However, teachers at school 3 expressed that 45-min lessons were adequate, showing the importance of individual adaptation.

### Children’s response

Most of the children liked the physically active academic lessons very much. Things they enjoyed include being physically active, being outside and the added variation to the school day. The finding that physical activity can increase children’s enjoyment at school is in line with other studies [20, 35]. It should be noted that statements from the children in the present study highlight the importance of variation within the physically active lessons. If the lessons become monotonous, for example including just running and the repetitive calculations, it becomes boring. Interventionists therefore need to understand the importance of developmentally appropriate, enjoyable and motivating physically active academic lessons.

### Lessons learned

In retrospect, we have found several factors that could be of interest for new schools implementing physically active academic lessons: **1)** Principals are the key to change in schools and it is important to support them as leaders of the implementation. Skilled external intervention partners could reduce principals’ work during implementation, but this should not result in more passive leadership and involvement by the principal. It is essential that principals are aware of the importance of facilitating the intervention by e.g., including physically active lessons into the curriculum and acknowledging the efforts of the intervention teachers. **2)** Teachers had a heavy workload during the startup, so a more gradual inclusion of the physically active lessons, e.g. once (not twice) a week, might be advisable. **3)** Teaching the teachers short, high-quality lessons could rapidly improve teachers’ self-efficacy for this teaching method. **4)** To be able to interact with other children in a positive way, children with concentration difficulties needed a thorough explanation of how to perform the tasks in the physically active lessons. **5)** Interventionists should consider differentiating the information and training given at each school. Some schools need more help than others. **6)** The teachers wanted to continue to use physically active academic lessons after the intervention period, but do them less frequently and with a shorter duration. Some wanted to choose their teaching method freely and did not want to have scheduled physically active lessons. Since this may hasten their retreat to old practices, the school leaders and teachers should discuss and share a cooperative plan for the implementation practice. **Finally**, including physical activity into the school’s overall strategies could make it easier to maintain focus on physical activity over time. This is also supported by Larsen, Samdal and Tjomsland [36]. Implementing physically active academic lessons could also act as a project for school development.

It has been found that teachers implementing educational changes need individual innovation-specific capacities [37], and to be successful in the long run such changes require determination, knowledge, time and effort at the school level, involving a strong leadership [38]. To increase the chances of lasting change, implementation focus must be maintained over years.

### Study limitations

Only 50% of the weekly teacher logs were received, and at one school, two out of three teachers did not provide any teacher logs. The implementation fidelity is therefore based on half of the intervention weeks. However, all teachers in the low-logging school participated in the interview after the intervention, and there was no reason to believe that the unreported periods differed significantly from the periods covered by the logs. Further, not all intervention teachers had the opportunity to participate in the group interview, so their experiences and opinions are missing. The intervention fidelity was based on subjective teacher reports with no objective control. Even though a small sample size (five intervention schools) limits the ability to generalise, the depth of information collected from both from both school leaders, teachers and children is a strength of the study. It should also be mentioned that other constructs in addition to those identified in the interview, may be important to implementation. An important next step would be testing the found implementation strategies in a real world setting.

## Conclusion

The use of physically active academic lessons was well received among school leaders, teachers and children, and they were accepted as a meaningful way to increase both learning, physical activity and health. This teaching method could be an effective way to increase physical activity in school without reducing academic time. Most teachers in the present study preferred a duration of 15–20 min for physically active lessons, meaning this method seems suitable for breaking up normal classroom teaching and reducing sedentary time.

To facilitate the implementation of physically active academic lessons in new schools, the importance of an engaged and motivated principal cannot be underestimated. Key actions by the principal include involving the teachers, setting priorities by e.g., adjusting the workload for the intervention teachers, acknowledging the teachers’ efforts, including physical activity into the school’s overall strategy, and maintaining the implementation focus over time. Introducing teachers to easily organized physically active lessons, and providing significant teacher support would lower the barriers to implementation.
